# Cloning and Characterization of a Putative Farnesoic Acid Omethyltransferase Gene from the Brown Planthopper, *Nilaparvata lugens*

**DOI:** 10.1673/031.010.10301

**Published:** 2010-07-13

**Authors:** Shuhua Liu, Chengwei Zhang, Baojun Yang, Jianhua Gu, Zewen Liu

**Affiliations:** ^1^ Key Laboratory of Monitoring and Management of Plant Disease and Insect, Ministry of Agriculture, Nanjing Agricultural University, Nanjing 210095, China; ^2^ The Service Center of Technology, Changzhou Entry-Exit Inspection and Quarantine Bureau, Changzhou 213022, China; ^3^ Rice Technology Research and Development Center, China National Rice Research Institute, Hangzhou 310006, China

**Keywords:** juvenile hormone synthesis, mRNA levels, ovary development

## Abstract

Juvenile hormone (JH) plays key roles in both metamorphosis and adult reproductive processes. Farnesoic acid *O*-methyltransferase (FAMeT) is thought to be an important enzyme in the JH biosynthetic pathway, catalyzing methylation of farnesoic acid (FA) to methyl farnesoate (MF). A full-length cDNA (*NlFAMeT*) encoding a 299 amino acid putative FAMeT was isolated from the brown planthopper, *Nilaparvata lugens* (Stal) (Hemiptera: Geometroidea), a major rice pest in many parts of Asia. *NlFAMeT* showed high amino acid identities (52–54%) with other insect FAMeTs. Although the *NlFAMeT* transcript was expressed highly in corpus allatum (CA) and brain (without CA), no correlation was found between *NlFAMeT* transcript and JH titers. Although only a low level of *NlFAMeT* transcript was detected in the ovary, a high level was found in the abdomen and should be in one or more tissues undefined in the abdomen. Also, *NlFAMeT* transcript had a positive change during the vitellogenesis in female adults. These data indicated that *NlFAMeT* might not be a key enzyme in JH synthesis in *N. lugens*, but that it may play an important role in the ovary development. It might also be important in some unknown process in a so-far unidentified tissue in the abdomen.

## Introduction

Juvenile hormones (JHs) are sesquiterpenoid compounds that are synthesized by the corpus allatum (CA) and are important in many pre- and postmetamorphic events in insects. JHs play major roles in the control of growth, development, metamorphosis, diapause and reproduction in insects (Riddiford et al. 2003). The regulation of JH titers is thus critical in the entire life of the insect. Neurosecretory cells in the brain release allatotropic and allatostatic factors that regulate the synthesis and secretion of JH (Stay 2000). Some important enzymes also play key roles in the synthesis and regulation of JH, such as JH methyltransferase and JH epoxidase (for a review, see Bellés et al. 2005).

The biosynthesis of JH can be divided into early and late phases: early phase being the production of farnesyl biphosphate (FBP) by the mevalonate pathway and late phase involving the conversion of FBP into JH precursors and eventually JH as the end product (Bellés et al. 2005). In the late phase in crustaceans, methyl farnesoate (MF) is the immediate precursor of JH III (Laufer et al. 1987). In crustaceans, MF may regulate juvenile characteristics in a manner similar to that of JH in insects. (Burtenshaw et al. 2008). Farnesoic acid *O*methyltransferase (FAMeT) is the enzyme that catalyzes the methylation of farnesoic acid (FA) to MF with the cofactor Sadenosyl-L-methionine (SAM) in crustaceans (Holford et al. 2004). The first study of FAMeT was in the crustacean *Metapenaeus ensis*, the sand shrimp (Gunawardene et al. 2001; 2002). The catalytic activity of FAMeT is thought to occur at a rate-limiting step in the JH biosynthetic pathway although its exact role is yet to be defined (Williamson et al. 2001).

Recently, the first putative FAMeT (CG10527) in insects was isolated from *Drosophila melanogaster*, although no detectable activity was found in the recombinant FAMeT expressed in a bacterial system (Burtenshaw et al. 2008). The orthologous sequences of *D. melanogaster* FAMeT are also found in other insect species, such as *Aedes aegypti* (XP001658262.1), *Anopheles gambiae* (XP_318631.4), *Apis mellifera* (XP_ 623146.1), *Melipona scutellaris* (CAM 35482.1), *Nasonia vitripennis* (XP_00 1599775.1) and *Tribolium castaneum* (XP974395.1). Here, the cloning of full-length cDNA encoding a putative FAMeT gene (*NlFAMeT*) is reported from the brown planthopper, *Nilaparvata lugens* (Stal) (Hemiptera: Geometroidea), a major rice pest in many parts of Asia, which causes a big loss in rice production in recent years. The tissue and developmental expressions of *NlFAMeT* were also included.

## Materials and Methods

### Experimental insects

Insects, *N. lugens*, were kept in laboratory cages at 25 ± 1° C, 70–80% RH, and a 16:8 L:D photoperiod. The developmental stages were synchronized at each larval molt. Corpora allata (CA), brain (without CA), fat body, ovary, accessory glands and midgut tissues were dissected from macropterous (long-winged) females (5th instar or adults) in phosphate buffered saline (PBS) treated with 0.1% diethylpyrocarbonate (DEPC) and stored at -70° C prior to use.

### Amplification of a putative FAMeT cDNA

When this work was started, the EST database of *N. lugens* (http://bphest.dna.affrc.go.jp/) was not available, so the RT-PCR technique with degenerate primers was used to clone the initial fragment. Total RNA was isolated by Trizol kit (Invitrogen, www.invitrogen.com). Synthesis of firststrand cDNAs was carried out according to the reverse transcriptase XL (AMV) (Takara, www.takara-bio.co.jp) protocol with oligo dT_18_. The first strand cDNA (1 µl) was used as a template for PCR. Degenerate primers, BP1 (GGNGTNT GYACNGGNATGGGNGC) and BP2 (CC NCCRTANGGDATRTARCANAC), were designed from the conserved regions of insect FAMeTs, which were GVCTGWGA and VCYIPYGG, respectively. The components of PCR were PCR reaction buffer containing 0.1 m*M* dNTP, 5 µ*M* each primer, and 1.0 unit of Ex-Taq DNA polymerase (Promega, www.promega.com) in a total volume of 20 µl. The thermal cycling condition was 95° C for 5 min followed by 35 cycles of 94° C for 45 s, 50° C for 1 min and 72° C for 1 min. The last cycle was followed by final extension at 72° C for 10 min. The amplified product was separated onto agarose gel and purified using the Wizard PCR Preps DAN Purification System (Promega). Purified DNA was ligated into the pGEM-T easy vector (Promega) and several independent subclones were sequenced from both directions. The sequence of the product was compared using Blast of the EST database of *N. lugens* (http://bphest.dna.affrc.go.jp/) and one clone (CNLHT2882) was found similar to FAMeT protein. The full-length cDNA was obtained by the RACE (rapid amplification of cDNA ends) technique according to the Smart Race kit (Clontech, www.clontech.com) protocol with genespecific primers (GSPs) for 5′-RACE (GCCACTGGAACCCAGGTAGCAG) and 3′-RACE (GGCAAAGTGGTGCCTTCGCATGG). In order to find out whether the sequence of C_NLHT2882 clone includes the complete 5′UTR (untranslated region), 5′-RACE was also carried out to identify the 5′-terminal region of the putative FAMeT gene.

### Quantitative real-time reverse transcriptase polymerase chain reaction (qRT-PCR)

The mRNA levels were measured by qRT-PCR using the One Step SYBR PrimeScript RT-PCR Kit (Takara). Total RNAs were treated by DNase I (Sigma-Aldrich, www.sigmaaldrich.com). qRT-PCR was performed in a 25 µl total reaction volume containing 5 ng of total RNA, 0.5 µl primer mix containing 10 µ*M* each of forward and reverse gene specific primers, 0.5 µl of Ex TaqTM HS (5 U/µl), 0.5 µl of PrimeScript RT Enzyme Mix, 12.5 µl of 2×One Step SYBR RT-PCR Buffer and 8.5 µl of H_2_O. Two types of negative controls were set up: non-template reactions (replacing total RNA by H_2_O) and minus reverse transcriptase controls (replacing PrimeScript RT Enzyme Mix by H_2_O). The qRT-PCR was done with the following cycling regime: initial incubation of 42° C for 5 min and 95° C for 10 s; 40 cycles of 95° C for 5 s, 60° C for 20 s, and 72 °C for 15 s. Standard curves were obtained using a ten-fold serial dilution of pooled total RNAs from 20 individuals. β-actin (EU179846) was used as an internal control (Liu et al. 2008). The mRNA expression was quantified in relation to the expression of β-actin. The primer pair of each gene was designed to amplify about 200 bp PCR products, which were verified by nucleotide sequencing. Only data that showed good efficiency (≥85%) and correlation coefficient (≥95%) were included in the analysis. Means and standard errors for each time point were obtained from the average of three independent sample sets. Gene specific primers for FAMeT and βactin were listed as: FAMeT-F: GCAAAGTCAGCAATCCGCAAGAAC; FAMeT-R: ACACCGTAGTGGGTGACAACGAATG; β-F: TGGACTTCGAGCAGGAAATGG; β-R: ACGTCGCACTTCATGATCGAG.

### JH titer determination

JH titers were determined as previously described (Liu et al. 2008). The GC-MS system consisted of a Hewlett Packard HP6890 series II gas Chromatograph and a mass selective detector (model 5973MS). 10 mg (about 6 individuals of the 5th instar female) *N. lugens* whole bodies were dried in Modulyod-230 Freeze Dryer (Thermo Electron) and were homogenized in 0.5 ml Hexane. The contents were centrifuged at 13,000 × g and 4° C for 10 min. The supernatant was dried using a stream of N_2_ gas and diluted to 25 µl in hexane. GC operating conditions: column HP-5, 25 m × 0.2 mm (length × diameter), film thickness 0.2 µm; column temperature programmed from 120° C (isothermal for 2 minutes) to 230° C (15° C/min); carrier gas helium, flow rate 40 ml/min; injector temperature 250° C; volume injected 1 µl. The standard JH III was purchased from Sigma-Aldrich. The overall mean for the standard JH III sample prepared at 150.0 ng/ml was 148.6 ng/ml (N = 35) with a standard deviation of 4.59 and a coefficient of variation (CV) of 2.4%. The reliability of the GC-MS methods was demonstrated by the coefficient of variation (CV ≤ 3.2%) on the standard JH III prepared at different concentrations (50–300 ng/ml).

**Figure 1.**
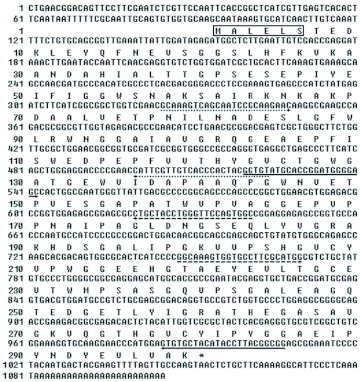
Nucleotide and deduced amino acid sequence of *NlFAMeT*. The positions of the primers used in the initial degenerate RT-PCR are shown by single lines with direction arrows. The primers used in rapid amplification of cDNA ends (RACE) are shown by the dashed lines with direction arrows. The primers used in quantitative real-time reverse transcriptase polymerase chain reaction (qRT-PCR) are shown by the dotted lines with direction arrows. A putative SANT domain profile is boxed. The stop codon is indicated by an asterisk (*). High quality figures are available online.

### Statistical analysis

Differences in mRNA levels or JH titers were analyzed by one-way ANOVA with at least three repeats. In all tests, 10–12 mg whole body weight was used, which included about 5–6 individuals of female adult, 6–8 individuals of the fifth instar female, or 10–12 individuals of the fourth instar larvae. For larvae under the fourth instar, larvae were pooled according to 1012 mg weight. Three repeats mean that at least three pools were used. Differences between treatment means were analysed using a least significant digit pair-wise comparison of means. The level of significance for results was set at p < 0.05.

## Results

### *NlFAMeT* cDNA

**Figure 2.**
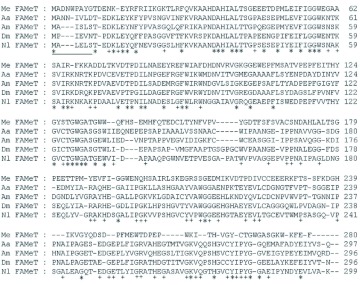
The alignment of amino acid sequence of *NlFAMeT* with the sequences of other insect FAMeTs. Numbers on the right side of the alignment indicate the position of residues in the sequence of each protein. Identical amino acids are indicated by asterisks (*). The amino acids that are identical in four insect FAMeTs, but not in *M. ensis* FAMeT, are indicated by plus signs (+). A putative SANT domain profile is underlined. *MeFAMeT* (*Metapenaeus ensis*, AAK28535.1), *AaFAMeT* (*Aedes aegypti*, XP_001658262.1), *AmFAMeT* (*Apis mellifera*, XP623146.1), *DmFAMeT* (*Drosophila melanogaster*, NP_6111544.1) and *NlFAMeT* (*Nilavarpata lugens*, FJ028722) are used in the alignment. High quality figures are available online.

RT-PCR and RACE techniques were used to clone the full-size *N. lugens* FAMeT (*NlFAMeT*) cDNA. Figure 1 shows the fulllength cDNA sequence with the deduced amino acid sequence above the nucleotide sequence (Genbank accession number, FJ028722). The sequence has an open reading frame (ORF) of 897 bp and 299 deduced amino acids. The deduced protein sequence of *NlFAMeT* showed 52%–54% identities to FAMeTs from *A. aegypti* (XP_001658262.1), *A. mellifera* (XP_623146.1), and *D. melanogaster* (NP_611544.1). *NlFAMeT* also showed 32% identity at the amino acid level to *M. ensis* FAMeT (AAK28535.1), the first studied FAMeT from crustaceans (Figure 2).

### Tissue expression of *NlFAMeT* mRNA

**Figure 3.**
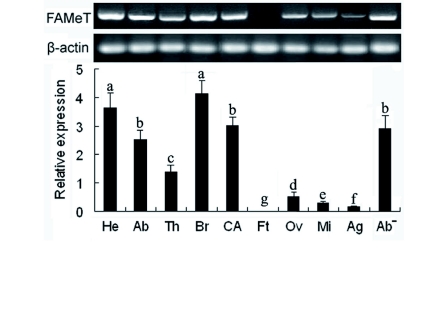
Tissue expression of the *NlFAMeT* mRNA in macropterous females detected by the quantitative real-time RT-PCR. (Above) The gel picture of qRT-PCR. The band for FAMet is 200 bp in length, and that for β-actin is 199 bp. (Below) The relative expression levels of *NlFAMeT* mRNA in different tissues. He, head (with CA); Ab, abdomen; Th, thorax; CA, corpus allatum; Br, brain (without CA); Ft, fatbody; Ov, ovary; Mi, midgut; Ag, accessory glands; Ab-, abdomen without Ft, Ov and Mi tissues. The data represent mean values ± SE of at least three repeats, normalized relative to β-actin transcript levels. Different lowercase letters above the columns indicate the significant differences at the p < 0.05 level. High quality figures are available online.

The *NlFAMeT* mRNA levels of different parts (head, thorax, and abdomen) and tissues (CA, brain, fatbody, ovary, accessory glands, and midgut) of macropterous females (1 day old) were determined by qRT-PCR (Figure 3). The head part showed the highest expression of *NlFAMeT* among three parts tested. Among all tissues tested, the brain (without CA) showed the highest expression level, and CA also showed very high expression. Low levels were detected in the ovary, midgut and accessory glands, and no trace was found in the fatbody. When the tissues, fatbody, ovary, and midgut were removed from the abdomen, the remaining parts (Ab ) also showed a high expression level of *NlFAMeT*, which indicated one or some tissues other than fatbody, ovary and midgut contained high levels of *NlFAMeT* mRNA.

**Figure 4.**
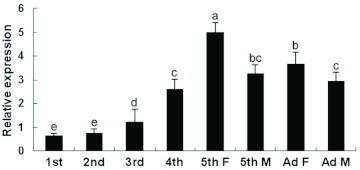
Developmental expression of the *NlFAMeT* mRNA in head parts of *Nilavarpata lugens*. 1 st, 1 st instar larvae; 2nd, 2nd instar larvae; 3rd, 3rd instar larvae; 4th, 4th instar larvae; 5th F, 5th instar female larvae; 5th M, 5th instar male larvae; Ad F, female adults (1 day old); Ad M, male adults (1 day old). The data represent mean values ± SE of at least three repeats. Different lowercase letters above the columns indicate the significant differences at the p < 0.05 level. High quality figures are available online.

### Developmental expression of *NlFAMeT* mRNA

The *NlFAMeT* mRNA levels of the head parts from larvae and macropterous adults were also determined (Figure 4). The samples were used from 1-day-old larvae in each instar and 1-day-old adults. In larvae, from 1st instar to 5th instar, the expression levels increased gradually and reached the peak in the 5th instar. Both in the 5th instar and in adults, the expression levels in female insects were significantly higher than those in male insects. In female insects, but not male insects, the expression level in the 5th instar larvae was obviously higher than that in adults.

### *NlFAMeT* mRNA levels and JH titers in 5th instar larvae

**Figure 5.**
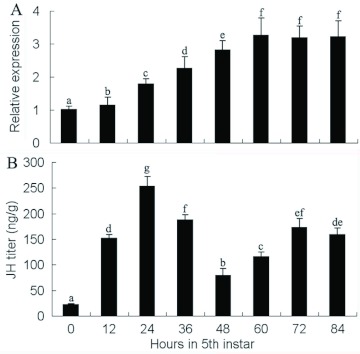
The dynamics of the relative *NlFAMeT* transcript levels (A) in corpus allatum (CA) and JH titers (B) in 5th instar macropterous females. The data represent mean values ± SE of at least three repeats. Different lowercase letters above the columns indicate the significant differences at the p < 0.05 level. High quality figures are available online.

In order to evaluate the relationship between *NlFAMeT* mRNA levels and JH titers in 5th instar larvae (macropterous female), *NlFAMeT* expression levels in CA and JH titers were determined with a 12 h interval. In the 5th instar macropterous female larvae, *NlFAMeT* mRNA levels increased gradually from its ecdysis and reached the peak at 60 h, which kept with little variation until the adult emergence (Figure 5A). The peak of JH titers existed at 24 h after the ecdysis of the 5th instar macropterous female larvae and had two bottom expressions at 0 h and 48 h (Figure 5B). These results showed that *NlFAMeT* mRNA levels and JH titers had no correlation in the 5th instar macropterous female larvae.

### *NlFAMeT* mRNA levels at different stages of ovarian development

**Figure 6.**
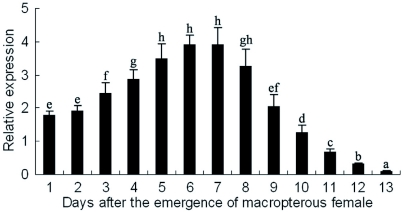
The dynamics of the relative *NlFAMeT* transcript levels in Ab- samples from macropterous female adults. The data represent mean values ± SE of at least three repeats. Different lowercase letters above the columns indicate the significant differences at the p < 0.05 level. High quality figures are available online.

In macropterous females of *N. lugens*, the vitellogenesis begins at the 2nd day of the emergency, peaks at the 7th day and terminates at the 14th day (Yi 2005). During this period, *NlFAMeT* mRNA levels of Ab- samples from macropterous females were determined each day. As shown in Figure 6, *NlFAMeT* mRNA levels increased gradually from the emergence of macropterous females and peaked at the 6th and 7th days, after which the expression levels decreased quickly and reached at a very low level at the 13th day. These results showed that the changes of *NlFAMeT* mRNA levels were in accordance with the vitellogenesis period.

## Discussion

Farnesoic acid *O*-methyltransferase (FAMeT) is the enzyme that catalyses the final step in the MF biosynthetic pathway in crustaceans (Wainwright et al. 1998; Wang et al. 1994). FAMeT was also present in the insect corpora allata. Variations in activity of the *O*-methyltransferase during development appear to be an important component in the regulation of JH biosynthesis in insects (Gunawardene et al. 2002). FAMeT is thought to catalyze the conversion of FA to MF using SAM as a cofactor. While this enzyme has been extensively studied in crustaceans, there is little known about its role in insects.

By RT-PCR and RACE techniques, a fulllength cDNA coding a putative FAMeT (*NlFAMeT*) was cloned from *N. lugens*. The deduced protein sequence showed high identities to other insect FAMeTs, which suggested that the *NlFAMeT* cDNA encodes a FAMeT or FAMeT-like protein. Although FAMeT is thought to catalyze the reaction using a SAM cofactor, the sequence in all organisms examined lacked the typical SAM binding motif, including *M. ensis* FAMeT (Figure 2), which is currently the only FAMeT that displays the activity *in vitro* (Gunawardene et al. 2002), suggesting FAMeT from insects, as well as from crustaceans, may be a novel SAM independent methyltransferase (Burtenshaw et al. 2008). A putative SANT (switching-defective protein 3, adaptor 2, nuclear receptor corepressor, and transcription factor IIIB) profile is present in the N-terminal region of *D. melanogaster* FAMeT, suggesting that protein-protein interactions might be required for functional structure and activity of insect FAMeT proteins (Figure 2). The recombinant FAMeT (rFAMeT) from *D. melanogaster* was expressed and purified in a bacterial system, but no activity was detected either with the rFAMeT alone or when added to a CA extract (Burtenshaw et al. 2008). In the laboratory, the heterologous expression of *NlFAMeT* in a bacterial system had been tried, but no positive results were obtained; so those expression data were not included in this report.

Although the first insect FAMeT was been isolated and characterized in *D. melanogaster*, the tissue expression of insect FAMeT mRNA is little known. Here, *NlFAMeT* was shown to be highly expressed in the head and abdomen, and the peak level was detected in the brain and CA, which indicated that the head and abdomen were the main sources of *NlFAMeT* transcript. In *D. melanogaster*, CA portion of the ring gland was identified as the important tissue highly expressing FAMeT (Burtenshaw et al. 2008). In the cockroach, *Diploptera punctata*, the rate limiting step controlling JH III biosynthesis in CA is catalyzed by FAMeT (Yagi et al. 1991). These results indicated CA is the main tissue expressing insect FAMeT transcript. Although the relatively high level of *NlFAMeT* transcript was detected in the abdomen, all tissues dissected from the abdomen only showed low levels. When these tissues were removed from the abdomen, the remaining part (Ab-) still showed a high level of *NlFAMeT* transcript, which indicated one or some unknown tissues contain high levels of *NlFAMeT* mRNA. Because of the insect's size, it is difficult to dissect more tissues completely from the abdomen, and the tissues leading to high levels of *NlFAMeT* transcript in the abdomen have not been found at present.

One key event is the clearing of JH that generally precedes the moult from the last larval stage to the pupal stage of holometabolous insects (Campbell et al. 2001). The very low JH titer at this time is generally achieved by the combined effect of reduced JH synthesis and scavenging by JH degrading enzymes (Roe and Venkatesh 1990). In *N. lugens*, with incomplete metamorphosis, the low JH titer is identified in the late stage of final instar (5th instar) (Dai et al. 2001). Some important enzymes are found to play key roles in the synthesis and regulation of JH (Bellés et al. 2005). To achieve the low JH titer in final instar, the key enzymes in the synthesis of JH will be lowly expressed and enzymes in the degradation of JH highly expressed, such as JH methyltransferase and JH esterase (Bellés et al. 2005; Dai et al. 2001). In insect species from *Lepidoptera*, the last steps include farnesoic acid synthesis by an aldehyde dehydrogenase, the conversion of FA to active JH (JH III) by C-10,11 epoxidation by a P450 monooxygenase and methylation of the carboxyl group by an S-adenosyl-L-methionine (SAM)-dependent methyltransferase (MTase). In species from *Orthoptera* and *Dictyoptera*, the last key steps include the methylation of FA to MF and the epoxidation of MF to active JH (Bellés et al. 2005; Shinoda and Itoyama 2003). Here, *NlFAMeT* transcript was highly expressed in the 5th instar larvae of *N. lugens*, which gradually increased from its ecdysis and kept at peak at its later stages (60–84 h). However, JH titers had the peak at 24 h and showed different changes during the 5th instar, compared to that of *NlFAMeT* mRNA levels. These results indicated FAMeT might not be a key enzyme in the synthesis of JH in this insect species, at least not in the 5th instar larvae.

Although *NlFAMeT* transcript level was low in the ovary, a high level was detected in some undefined tissues in the abdomen. And *NlFAMeT* transcript levels increased when then vitellogenesis began at the 2nd day and peaked at 6th–7th day after the emergence of macropterous females, which also was the peak of vitellogenesis. After the peak of vitellogenesis, *NlFAMeT* transcript levels decreased and reached a very low level when vitellogenesis terminated on the 13th day. These data indicated *NlFAMeT* might play important roles in the development of the insect ovary. In the shrimp, *M. ensis*, FAMeT mRNA is expressed throughout ovarian maturation in the nerve and eyestalk, suggesting a possible role of FAMeT in the regulation of reproduction (Gunawardene et al. 2002).

## Abbreviations

FAMeT: farnesoic acid *O*-methyltransferase;
FA: farnesoic acid;
MF: methyl farnesoate;
CA: corpus allatum;
JH: juvenile hormone;
SAM: Sadenosyl-L-methionine;
FBP: farnesyl biphosphate;
PCR: polymerase chain reaction;
qRT-PCR: Quantitative real-time reverse transcriptase polymerase chain reaction;
RACE: rapid amplification of cDNA ends
